# Supplementary material for: Baseline Characterization of the Gut Microbiota of Field and Colony Populations of Phlebotomus tobbi and Preliminary Assessment of the Anti-Leishmanial Activity of Cultivable Bacteria
Source: Pathogens. 2026 Jun 23;15(7):658. doi: 10.3390/pathogens15070658 (PMC13416292; doi:10.3390/pathogens15070658)
Supplement: Supplementary file 1 [file pathogens-15-00658-s001.zip › pathogens-4357168-File S1/Heatmap/4.order/heatmap_without_sample_clustering.pdf]

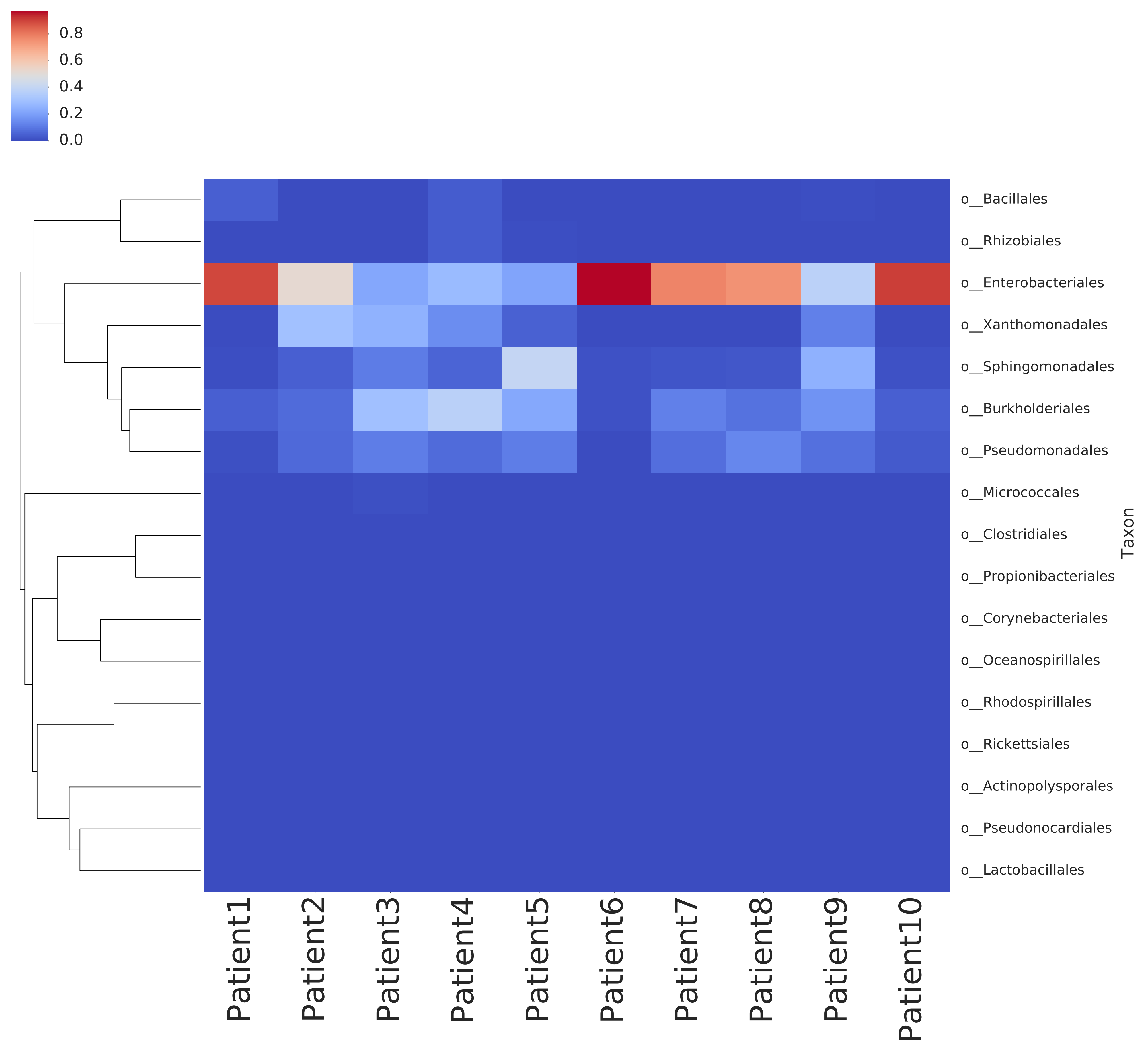
0.8
0.6
0.4
0.2
0.0
o__Bacillales
o__Rhizobiales
o__Enterobacteriales
o__Xanthomonadales
o__Sphingomonadales
o__Burkholderiales
o__Pseudomonadales
o__Micrococcales
o__Clostridiales
o__Propionibacteriales
o__Corynebacteriales
o__Oceanospirillales
o__Rhodospirillales
o__Rickettsiales
o__Actinopolysporales
o__Pseudonocardiales
o__Lactobacillales
Taxon
Patient1
Patient2
Patient3
Patient4
Patient5
Patient6
Patient7
Patient8
Patient9
Patient10
